# Supplementary figures and images for: RIPK1 regulates β-cell fate via actions on gene expression and kinase signaling in a mouse model of β-cell self-reactivity
Source: Cell Death Dis. 2026 Feb 12;17(1):220. doi: 10.1038/s41419-026-08471-0 (PMC12920666; doi:10.1038/s41419-026-08471-0)

Figure 1B

Phospho-RIPK1 (S166)

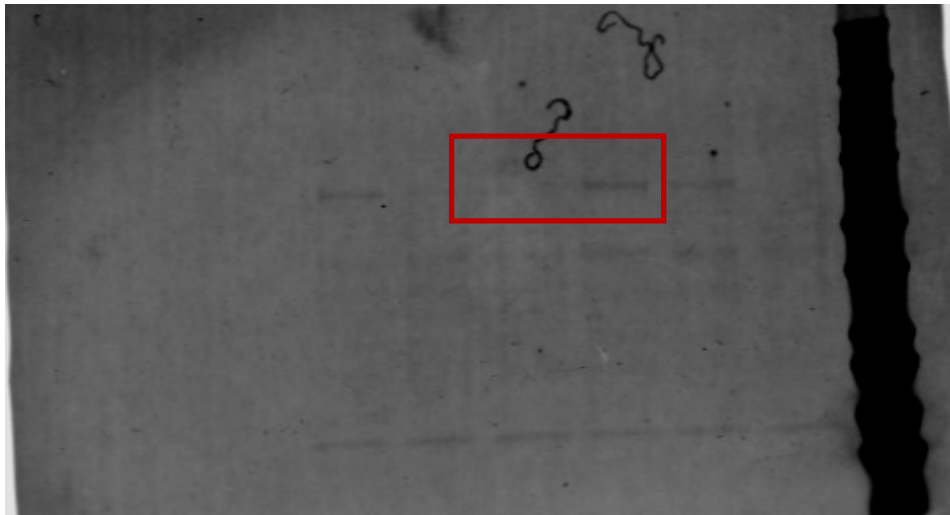

Total RIPK1

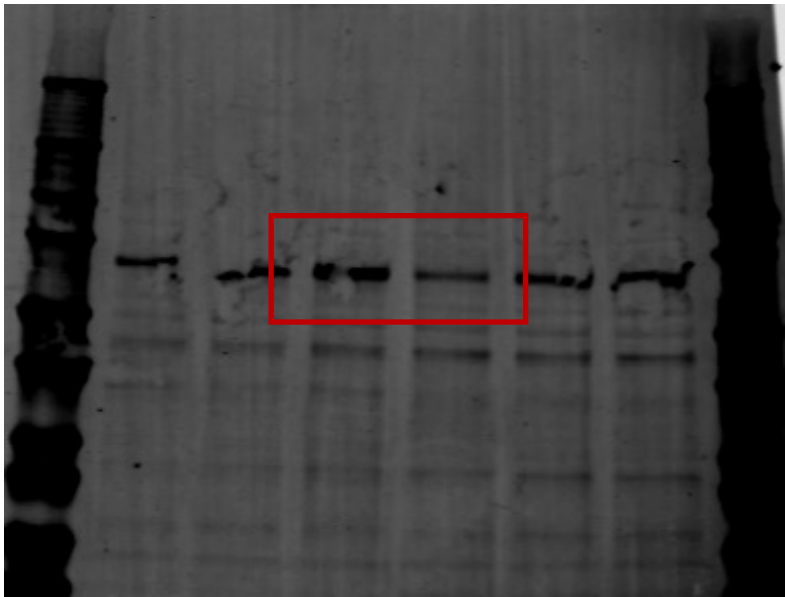

Figure 1C

Phospho-RIPK1 (S166)

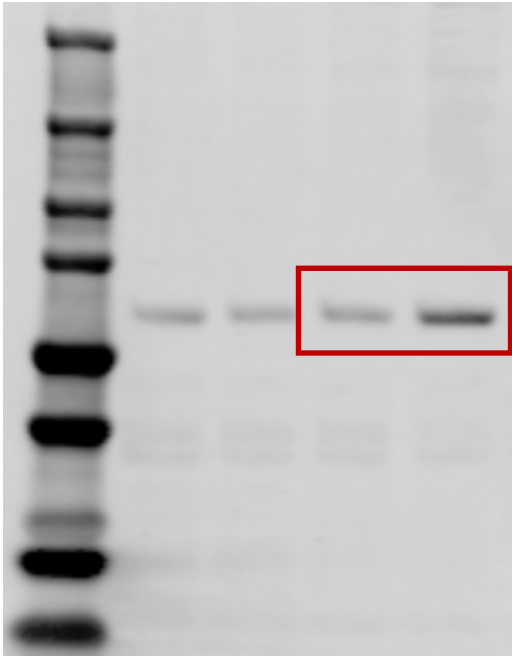

Total RIPK1

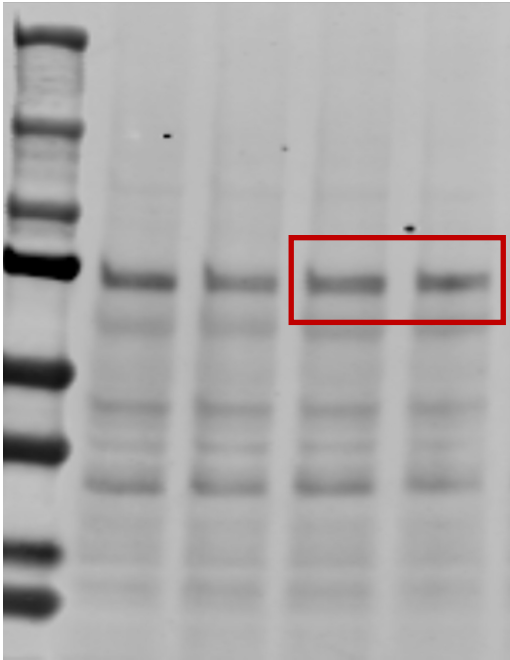

Figure 1D

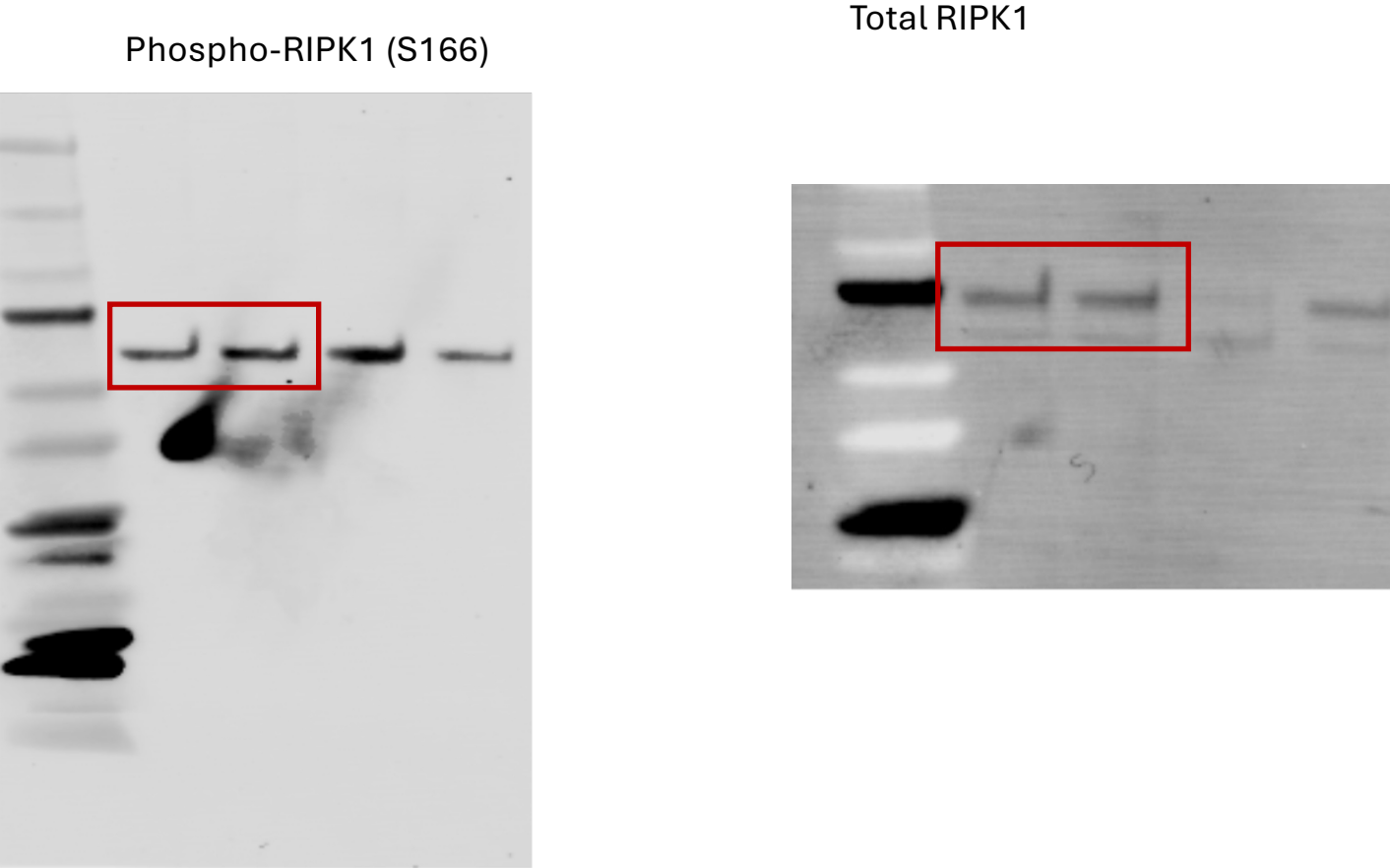

Figure 2C

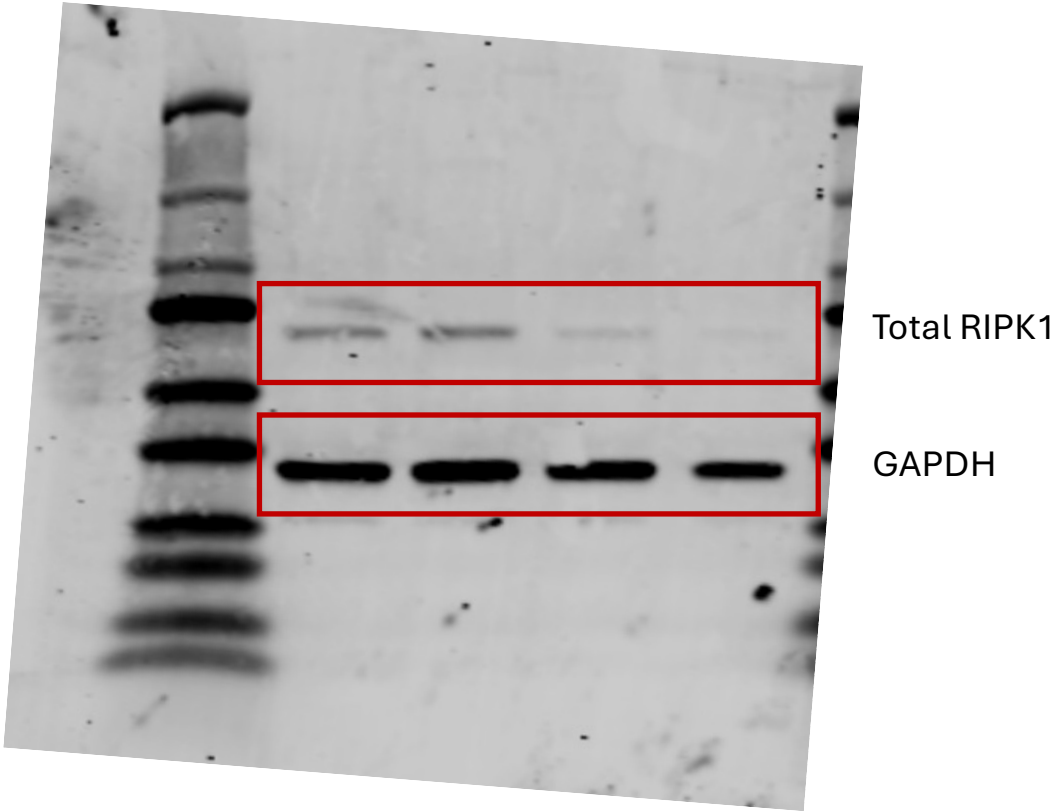

Figure 3A

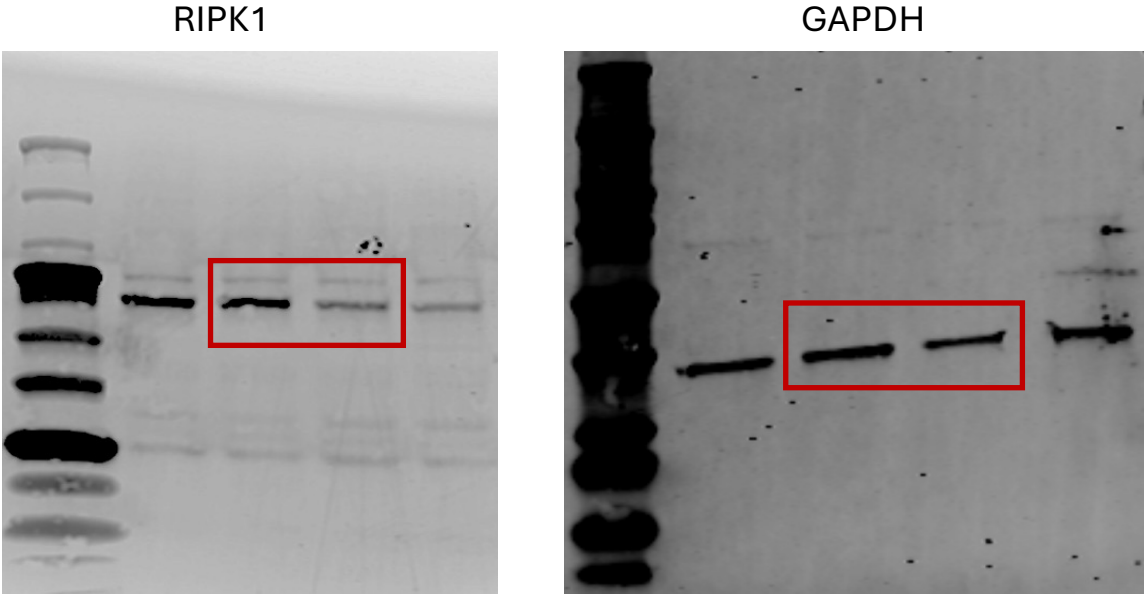

Supplement: Supplementary file 1 — Immunoblot Images [file 41419_2026_8471_MOESM1_ESM.pdf]
